# Regional Histopathology and Prostate MRI Positivity: A Secondary Analysis of the PROMIS Trial

**DOI:** 10.1148/radiol.220762

**Published:** 2022-12-13

**Authors:** Vasilis Stavrinides, Joseph M. Norris, Solon Karapanagiotis, Francesco Giganti, Alistair Grey, Nick Trahearn, Alex Freeman, Aiman Haider, Lina María Carmona Echeverría, Simon R. J. Bott, Louise C. Brown, Nicholas Burns-Cox, Timothy J. Dudderidge, Ahmed El-Shater Bosaily, Maneesh Ghei, Alastair Henderson, Richard G. Hindley, Richard S. Kaplan, Robert Oldroyd, Chris Parker, Raj Persad, Derek J. Rosario, Iqbal S. Shergill, Mathias Winkler, Alex Kirkham, Shonit Punwani, Hayley C. Whitaker, Hashim U. Ahmed, Mark Emberton

**Affiliations:** Division of Surgery and Interventional Science, University College London, Charles Bell House, 43-45 Foley St, London W1W 7TS, UK; The Alan Turing Institute, London, UK, Departments of Urology; Division of Surgery and Interventional Science, Departments of Urology; University College London, Charles Bell House, 43-45 Foley St, London W1W 7TS, UK; The Alan Turing Institute, London, UK, University College London Hospitals NHS Foundation Trust, London, UK; Medical Research Council Biostatistics Unit, University of Cambridge, Cambridge, UK; Division of Surgery and Interventional Science, Radiology; Division of Surgery and Interventional Science, Departments of Urology; Computational Pathology Group, Institute of Cancer Research, Sutton, London, UK; Pathology; Division of Surgery and Interventional Science, Pathology; Department of Urology, Frimley Health NHS Foundation Trust, London, UK; Medical Research Council Clinical Trials Unit; Department of Urology, Taunton & Somerset NHS Foundation Trust, Taunton, UK; Department of Urology, University Hospital Southampton NHS Foundation Trust, Southampton, UK; Department of Radiology, Royal Free London NHS Foundation Trust, London, UK; Department of Urology, Whittington Health NHS Trust, London, UK; Department of Urology, Maidstone & Tunbridge Wells NHS Trust, Tunbridge Wells, UK; Department of Urology, Hampshire Hospitals NHS Foundation Trust, UK; Medical Research Council Clinical Trials Unit; Public and patient representative, Nottingham, UK; Department of Academic Urology, The Royal Marsden NHS Foundation Trust, Sutton, UK; Department of Urology, North Bristol NHS Trust, Bristol, UK; Department of Urology, Sheffield Teaching Hospitals NHS Foundation Trust, Sheffield, UK; Department of Urology, Wrexham Maelor Hospital NHS Trust, Wrexham, UK; Department of Urology, Imperial College Healthcare NHS Trust, London, UK, and Imperial Prostate, Division of Surgery, Department of Surgery & Cancer, Faculty of Medicine, Imperial College London, London, UK; Radiology; Division of Surgery and Interventional Science, Centre for Medical Imaging, Radiology; Division of Surgery and Interventional Science; Department of Urology, Imperial College Healthcare NHS Trust, London, UK, and Imperial Prostate, Division of Surgery, Department of Surgery & Cancer, Faculty of Medicine, Imperial College London, London, UK; Division of Surgery and Interventional Science, Departments of Urology

## Abstract

**Background:**

The effects of regional histopathologic changes on prostate MRI scans have not been accurately quantified in men with an elevated prostate-specific antigen (PSA) level and no previous biopsy.

**Purpose:**

To assess how Gleason grade, maximum cancer core length (MCCL), inflammation, prostatic intraepithelial neoplasia (PIN), or atypical small acinar proliferation within a Barzell zone affects the odds of MRI visibility.

**Materials and Methods:**

In this secondary analysis of the Prostate MRI Imaging Study (PROMIS; May 2012 to November 2015), consecutive participants who underwent multiparametric MRI followed by a combined biopsy, including 5-mm transperineal mapping (TPM), were evaluated. TPM pathologic findings were reported at the whole-prostate level and for each of 20 Barzell zones per prostate. An expert panel blinded to the pathologic findings reviewed MRI scans and declared which Barzell areas spanned Likert score 3–5 lesions. The relationship of Gleason grade and MCCL to zonal MRI outcome (visible vs nonvisible) was assessed using generalized linear mixed-effects models with random intercepts for individual participants. Inflammation, PIN, and atypical small acinar proliferation were similarly assessed in men who had negative TPM results.

**Results:**

Overall, 161 men (median age, 62 years [IQR, 11 years]) were evaluated and 3179 Barzell zones were assigned MRI status. Compared with benign areas, the odds of MRI visibility were higher when a zone contained cancer with a Gleason score of 3+4 (odds ratio [OR], 3.1; 95% CI: 1.9, 4.9; *P* < .001) or Gleason score greater than or equal to 4+3 (OR, 8.7; 95% CI: 4.5, 17.0; *P* < .001). MCCL also determined visibility (OR, 1.24 per millimeter increase; 95% CI: 1.15, 1.33; *P* < .001), but odds were lower with each prostate volume doubling (OR, 0.7; 95% CI: 0.5, 0.9). In men who were TPM-negative, the presence of PIN increased the odds of zonal visibility (OR, 3.7; 95% CI: 1.5, 9.1; *P* = .004).

**Conclusion:**

An incremental relationship between cancer burden and prostate MRI visibility was observed. Prostatic intraepithelial neoplasia contributed to false-positive MRI findings.

ClinicalTrials.gov registration no. NCT01292291

In the human prostate, an MRI signal is generated by biologic processes with microstructural implications. For example, MRI-visible clinically significant cancer (csCa) is associated with increased cellularity and decreased luminal density, whereas false-positive findings are considered a byproduct of nonmalignant microenvironmental perturbations, such as inflammation (1–3). In men without prior biopsy and a raised prostate-specific antigen (PSA) level, a quantitative understanding of the relationship between regional pathologic changes and MRI positivity is a prerequisite for distinguishing true-positive from false-positive findings and mitigating unnecessary MRI-directed sampling (4). Unfortunately, because it is particularly difficult to comprehensively capture all possible MRI phenotypes in unperturbed prostates, many studies are afflicted by selection, spectrum, or sampling bias.

We recently studied a well-interrogated, biopsy-naive population from the Prostate MRI Imaging Study (PROMIS) to lay out a clinically useful distinction between true-positive and false-positive MRI findings by using readily available radiologic scores, PSA density, lesion volume, and diffusion restriction metrics (5,6). In brief, PROMIS was a multicenter paired cohort study that investigated the diagnostic accuracy of multiparametric MRI versus systematic transrectal US-guided biopsy against a reference standard (transperineal mapping [TPM], a highly accurate sampling technique where the prostate is sampled every 5 mm). The study proved that, in men with a raised PSA level and suspected cancer, multiparametric MRI helped detect more clinically significant disease with fewer needle deployments, whereas transrectal US biopsies missed up to half of significant tumors. At the same time, multiparametric MRI demonstrates 5% fewer insignificant cancers compared with transrectal US-guided sampling. The blinded design of the study (ie, MRI interpretation and combined TPM–transrectal US biopsy were independent) and the application of such a rigorous reference standard in previously biopsy-naive men give PROMIS its unique advantage, which is its relative freedom from spectrum, selection, and sampling biases. Because of the study’s uniqueness, it might be helpful for the reader to review its original design (see also Fig S1) (5).

The purpose of the work presented here was to assess how Gleason grade, maximum cancer core length (MCCL), inflammation, prostatic intraepithelial neoplasia (PIN), or atypical small acinar proliferation within a Barzell zone affects the odds of MRI visibility. In the process, we propose a Barzell zone–based framework of MRI positivity and demonstrate a simple method for aligning biopsy findings to MRI results that, despite its coarseness, could be useful in settings where computer-based registration of MRI to histopathologic sections is impossible.

## Materials and Methods

### Participants and Data

In total, 576 biopsy-naïve men with an elevated PSA level (≤15 ng/mL) were recruited in the original multicenter PROMIS study (ClinicalTrials.gov registration no. NCT01292291) from May 2012 to November 2015 (5). Ethics committee approval for PROMIS was originally granted by National Research Ethics Service Committee London (reference 11/LO/0185) and all participants provided written informed consent. Data ana-lyzed for this study were provided by a third party. Requests for data should be directed to the provider indicated in the Acknowledgments. Briefly, all participants underwent prebiopsy 1.5-T multiparametric MRI followed by a combined biopsy procedure under general anesthetic (5-mm TPM followed by standard systematic transrectal biopsy) performed by clinicians blinded to imaging findings. Standard reporting within PROMIS included age, presenting PSA level, and per-participant overall TPM pathologic findings designated by a uropathologist after considering global prostate cancer burden from the Gleason score and MCCL, according to International Society of Urological Pathology and UK standards (7). Overall TPM pathologic findings were used in PROMIS as the “ground truth” and resulted in classification of all prostates according to four well-established University College London definitions: (*a*) no cancer, (*b*) insignificant cancer (Gleason score of 3+3 with MCCL up to 4 mm), (*c*) definition 2 csCa (any Gleason score ≥3+4 and/or any MCCL ≥4 mm), and (*d*) definition 1 csCa (any Gleason score ≥4+3 and/or any MCCL ≥6 mm).

In a previous article, we considered a subgroup of consecutive PROMIS participants recruited only at University College London (6). This prior work dealt with clinical-radiologic characteristics (eg, PSA density and apparent diffusion coefficient) that distinguish MRI true- and false-positive findings, using prostate-level TPM results as a reference standard. In the current article, we quantify the direct impact of regional prostate pathologic changes on MRI visibility because, for consecutive PROMIS nonpilot participants, TPM pathologic findings were also reported per Barzell zone. The 20-zone modified Barzell scheme has been described previously and has been used in University College London trials (Fig S1) (8). Per-zone reporting included Gleason grading and MCCL information whenever cancer was detected; whereas, in noncancerous zones, the presence of inflammation, PIN, and atypical small acinar proliferation was reported in a binary fashion (present or not present). From a radiologic standpoint, apart from prostate volume (calculated at MRI using the ellipsoid formula) and overall per-prostate Likert scores for underlying csCa, information was additionally recorded at the lesion level (including per-sequence Likert scores, location, volume, and apparent diffusion coefficient).

### Consensus Alignment of Modified Barzell Zones to Multiparametric MRI Lesions (Likert Score ≥3)

Completely anonymized MRI scans were retrieved and randomly reviewed by a multidisciplinary panel consisting of a uroradiologist (F.G., with 8 years of experience in prostate MRI reporting), a urologist (A.G., with 10 years of experience in prostate intervention and MRI interpretation), and two uropathologists (A.F. and A. Henderson, with 20 and 6 years of experience in uropathology) who were blinded to TPM findings. After assessing each MRI scan separately (T2-weighted, apparent diffusion coefficient mapping, long *b* value, and dynamic contrast-enhanced sequences) and using a Barzell zone map as a guide, the panel was asked to declare by consensus which zones were “MRI-visible” (ie, spanned by a lesion with a Likert score ≥3) and which were “nonvisible.” If an MRI lesion covered more than one Barzell zone, then the panel was also asked to declare which zone “aligned best” with the lesion and designate its “nonvisible” counterpart in the most appropriate distant area (mirror position whenever possible) (9). After these steps and once the panel had finished assigning an MRI outcome to all zones, TPM pathologic findings were revealed such that lesions spanning at least one MRI-visible csCa-containing zone (according to University College London definitions) were deemed true positive, while lesions that did not span any such zones were considered false-positive.

### Statistical Analysis

Continuous and categorical characteristics were summarized using simple statistics such as means, medians with IQRs, and proportions. Nonparametric tests (Wilcoxon and Kruskal-Wallis analysis of variance) were used to detect between-group differences. To investigate the relationship between Barzell zone pathologic findings and the odds of that same zone being declared MRI-visible by the expert panel, we used mixed-effects logistic regression models with a binary outcome (visible or nonvisible zone) and pathologic variables as predictors (eg, Gleason grade, MCCL, etc). Because there were 20 Barzell zones per participant, we included random intercepts for individual participants to account for within-participant correlation and scrutinized this approach against a generalized linear model with fixed predictors only. The final model selection was based on the Akaike information criterion. R version 4.1.2 (The R Foundation; https://www.r-project.org) was used for all analyses, and P values were considered indicative of a statistically significant difference at the .05 level.

## Results

### Participant Characteristics Stratified by per-Prostate Gleason Grade

**Figure 1:**
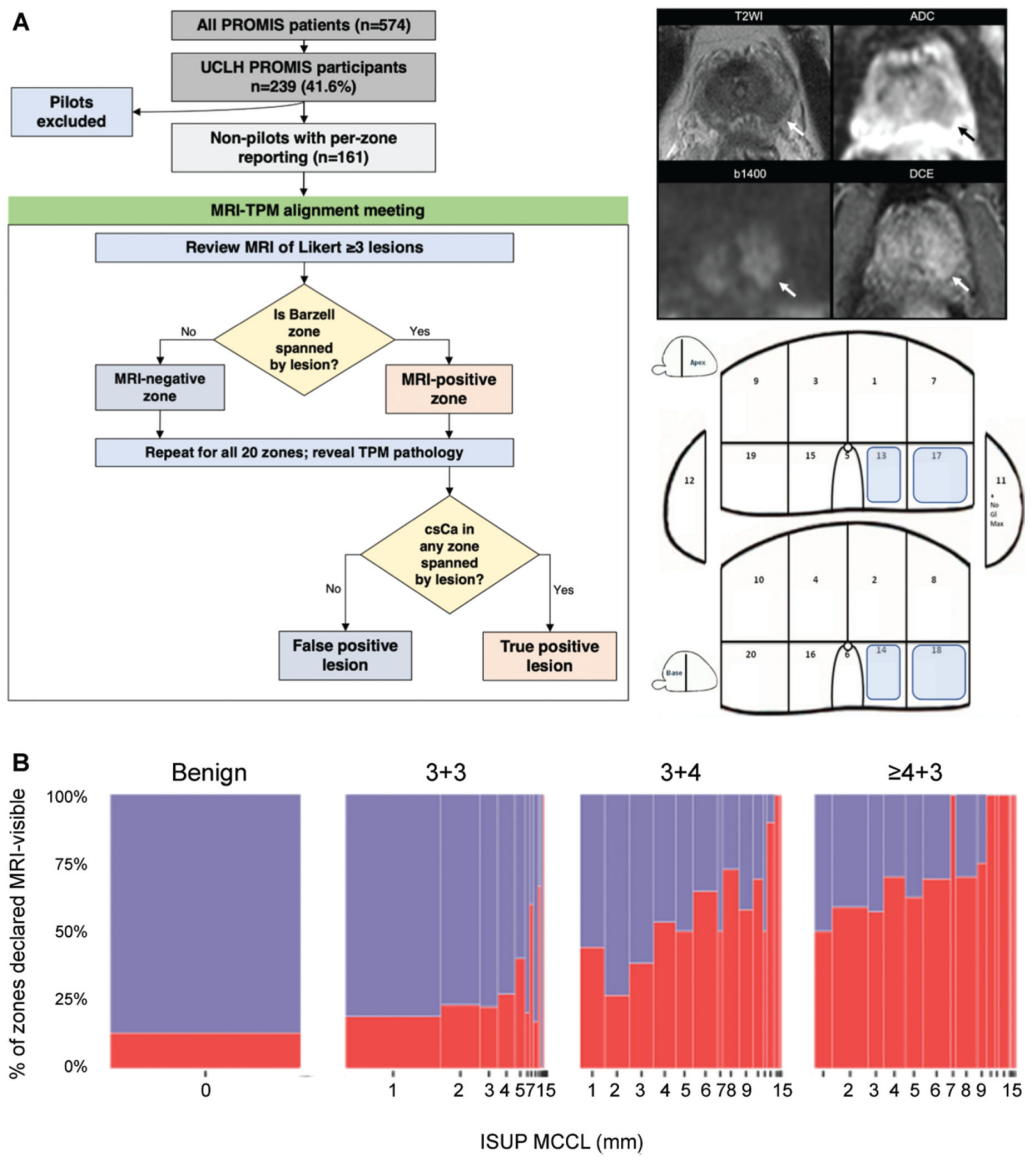
**(A)** Flowchart shows participant population and MRI-transperineal mapping (TPM) alignment. The study included 161 nonpilot Prostate MRI Imaging Study (PROMIS) participants from University College London Hospitals (UCLH) who underwent multiparametric MRI followed by a combined biopsy procedure and detailed per-zone recording of Gleason grade and maximum cancer core length (MCCL) per the International Society of Urological Pathology (ISUP) definition. A consensus multidisciplinary panel, blinded to TPM findings, reviewed MRI scans and aligned any lesions with a Likert score greater than or equal to 3 to specific Barzell zones before the pathologic status was revealed. For example, the left peripheral zone lesion shown on the right was aligned to zones 13,14,17, and 18. **(B)** Spineplots show the percentage of zones deemed visible at MRI according to the overall Gleason score at TPM. The consensus panel determined the MRI positivity of 3179 zones; this was slightly less than the expected 3220 (161 participants x 20 zones) due to small prostate size in five men, which prevented full sampling of all Barzell zones. Of 3179 zones, 2516 were benign and, of those with cancer, 301 had a Gleason score of 3+3, 271 had a Gleason score of 3+4, and 91 had a Gleason score greater than or equal to 4+3. In total, 595 zones were MRI-positive (18.7%), although the proportion that were MRI-visible rose with increasing Gleason grade and with each additional millimeter of MCCL, motivating a zonal pathologic finding-based model of MRI positivity. ADC = apparent diffusion coefficient, b1400 = b value of 1400, csCa = clinically significant cancer, DCE = dynamic contrast-enhanced, T2WI = T2-weighted imaging.

**Figure 2:**
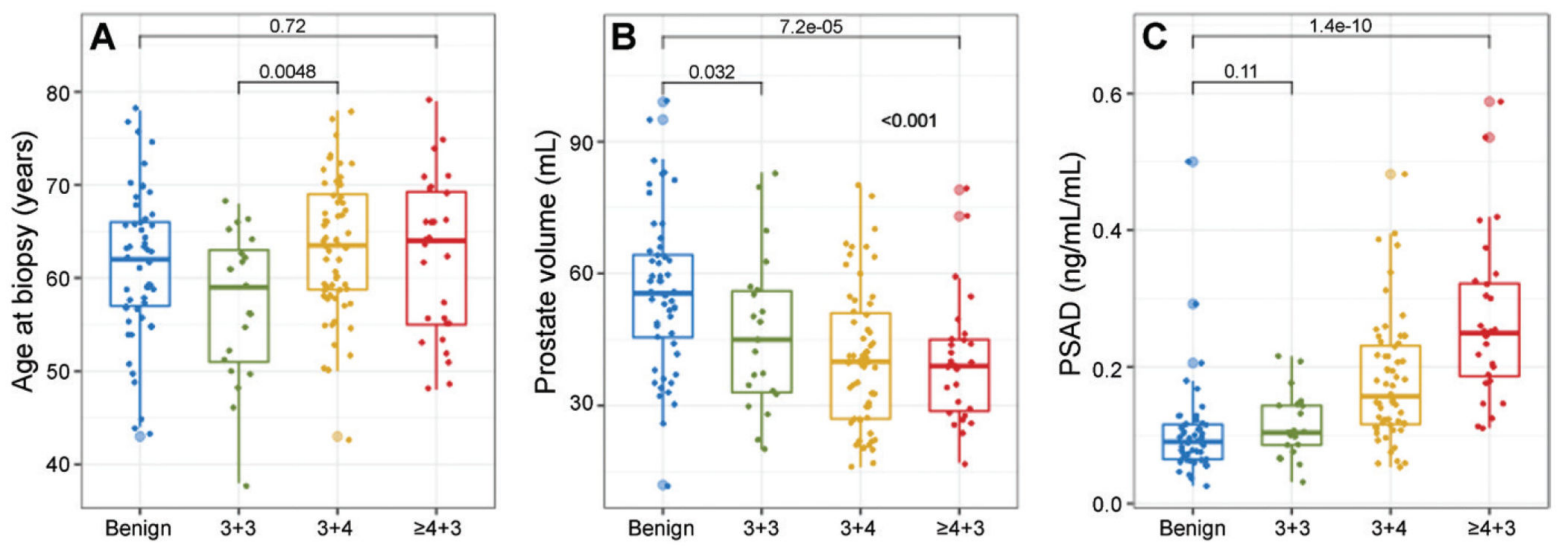
**(A)** Boxplot shows a moderate age difference between Gleason score groups (*P* = .04, Kruskal-Wallis analysis of variance) that was primarily driven by the lower median age of the Gleason 3+3 group (*n* = 21). **(B)** Boxplot shows men with an overall Gleason score greater than or equal to 4+3 at transperineal mapping (TPM) had low prostate volumes compared with other groups (*P* < .001, Kruskal-Wallis analysis of variance and adjusted pairwise comparisons). **(C)** Boxplot shows men with an overall Gleason score greater than or equal to 4+3 at TPM had high prostate-specific antigen (PSA) density (PSAD) (*P* < .001, Kruskal-Wallis analysis of variance and adjusted pairwise comparisons) and low prostate volume (as shown in **B**) compared with other groups. In men who were TPM-negative, this relationship was reversed; prostate volume was highest and PSA density lowest. These findings imply the existence of two distinct pathologic states in biopsy-naive men that, although both manifest as an elevated PSA level indicating the need for biopsy, differ in terms of the mechanism generating the increase in PSA.

Among the 576 men in the PROMIS trial, a subset of participants from a single institution, University College London Hospitals, were considered. Among those, 161 nonpilot participants met the criteria for this secondary analysis (median age, 62 years [IQR, 11 years]), while 78 pilot participants were excluded (Fig 1A). Baseline age, prostate volume, PSA level, and PSA density are shown in Table 1 for the entire cohort, as well as for groups stratified by overall Gleason score at TPM as designated by a uropathologist. Although the four Gleason grade groups were not substantially different in terms of age at first biopsy, pairwise comparisons revealed an important association of PSA density and prostate volume with grade (Fig 2). The largest difference in PSA density and prostate volume was observed between men with Gleason scores greater than or equal to 4+3 and those with no cancer at TPM (*P* < .001, Wilcoxon test), while men with Gleason scores of 3+3 and 3+4 had PSA density and prostate volume values between those two extremes.

### Zonal Gleason Grade and MCCL as Predictors of MRI Positivity (All Participants)

The consensus panel workflow is presented in Figure 1A. Of 3179 zones reviewed, 2516 (79.1%) were benign, 301 (9.5%) were cancerous with a Gleason score of 3+3, 271 (8.5%) had a Gleason score of 3+4, and 91 (2.9%) had a Gleason score greater than or equal to 4+3 (4+3 [n = 73], 4+4 [*n* = 11], 4+5 [*n* = 6], and 3+5 [*n* = 1]). The panel concluded that 595 of 3179 zones were MRI-positive (18.7%) and this proportion clearly depended on cancer burden (Fig 1B). MRI-positive zones included 319 of 2516 (12.7%) that were benign, 69 of 301 (22.9%) with a Gleason score of 3+3, 144 of 271 (53.1%) with a Gleason score of 3+4, and 63 of 91 (69%) with a Gleason score greater than or equal to 4+3 (4+3 [49 of 73], 4+4 [7 of 11], 4+5 [6 of 6], and 3+5 [1 of 1]). The proportion of MRI-positive cancerous zones increased as MCCL increased, regardless of Gleason grade (Fig 1B).

**Figure 3:**
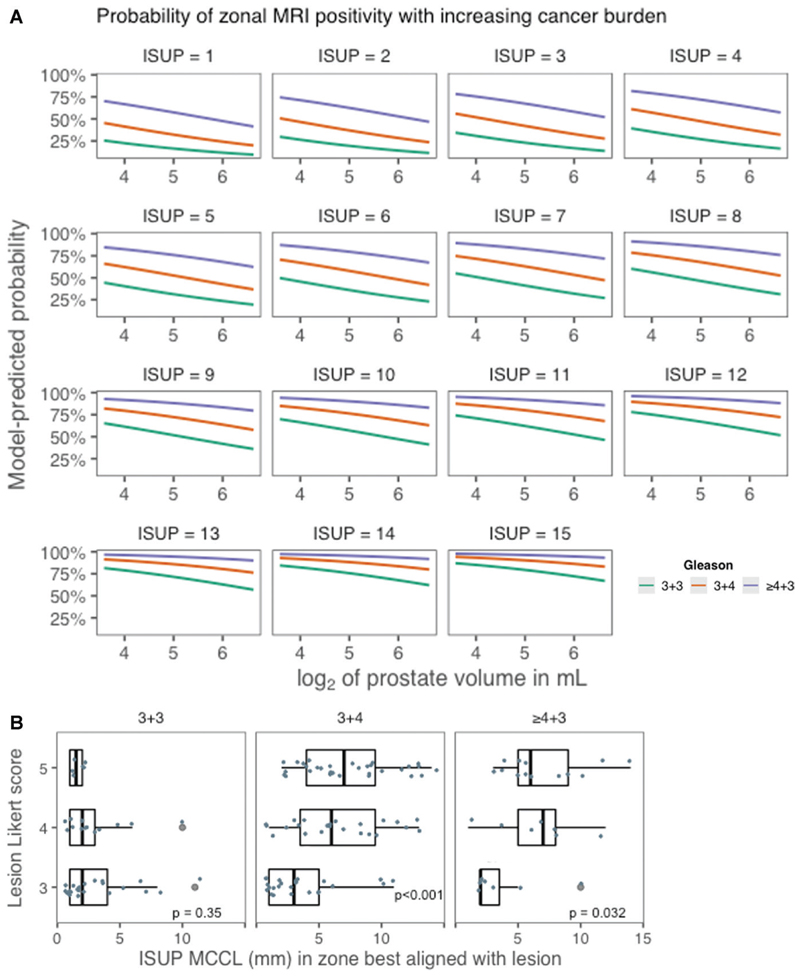
**(A)** Graphs show the predicted probabilities of a Barzell zone being visible at MRI when using the zonal Gleason grade, maximum cancer core length (MCCL), and binary logarithm (log_2_) of prostate volume (in milliliters) as predictors in a mixed model with random intercepts for participants (3179 zones in 161 men). The probability progressively increases with every increment in Gleason grade or MCCL, while prostate volume increase has the opposite effect. **(B)** Horizontal boxplots show the relationship between Likert scores and cancer burden. After selecting the Barzell zones spanning a specific lesion, the expert panel also agreed on a single zone having the “best” alignment. In total, 115 cancerous zones best-aligned with a lesion (index or secondary), and their pathologic classification against Likert scoring is shown. Greater MCCL was associated with higher radiologic scores (Likert score 4-5), particularly when Gleason pattern 4 was present (*P* < .05, Kruskal-Wallis). An MCCL less than approximately 5 mm was mostly associated with Likert 3 lesions (the main arena of true- and false-positive MRI distinction), regardless of cancer grade. ISUP = International Society of Urological Pathology.

Based on these observations, mixed-effects logistic regression models with random intercepts for individual participants and zonal MRI positivity as a binary outcome (visible or nonvisible) were fitted to the data. The final mixed model included the zonal Gleason grade, International Society of Urological Pathology MCCL (in millimeters), and binary logarithm of prostate volume (in milliliters) as predictors, and it performed better than intercept-only baseline models or models with each predictor alone (Table 2, Table S1). Gleason scores of 3+4 and greater than or equal to 4+3 were both significantly associated with outcome (*P* < .001), increasing the likelihood of a zone being MRI-positive by threefold (odds ratio [OR], 3.1; 95% CI: 1.9, 4.9; *P* < .001) and almost ninefold (OR, 8.7; 95% CI: 4.5, 17; *P* < .001), respectively. MCCL was also a predictor of zonal MRI positivity, with each additional millimeter corresponding to an OR of 1.24 (95% CI: 1.15, 1.33; *P* < .001) regardless of Gleason grade. Finally, in line with our initial observations on prostate volume, every volume doubling was associated with reduced odds of zonal MRI positivity (OR, 0.7; 95% CI: 0.5, 0.9; *P* = .02). The model-predicted probabilities of zonal MRI positivity are presented in Figure 3B for different combinations of Gleason grade, MCCL, and prostate volume.

### Zonal Inflammation, PIN, and Atypical Small Acinar Proliferation as Predictors of False MRI Positivity (TPM-Negative Group)

Mixed-effects logistic regression models were fitted with random intercepts for participants who had negative TPM results (*n* = 52). In these models panel-designated zonal MRI visibility was again a binary outcome, whereas inflammation, PIN, or atypical small acinar proliferation status within the zone was represented as the linear combination of three binary predictors (present vs not present). In an initial model including all three binary variables (Table 3), only PIN was a predictor of zonal MRI positivity (OR, 3.2; 95% CI: 1.3, 8.1; *P* = .01). After successive model fitting, a final reduced mixed model indicated that, in TPM-negative prostates, the presence of PIN in a Barzell zone almost quadrupled its odds of being visible at MRI (OR, 3.7; 95% CI: 1.5, 9.1; *P* = .004) (Table 3, Table S1).

## Discussion

In this study, we quantified the impact of prostatic pathologic changes on regional MRI visibility in men undergoing their first prostate-specific antigen–triggered biopsy. We found the presence of a Gleason grade 4 component substantially increases the odds of a Barzell zone being MRI-positive compared with benign tissue (odds ratio [OR], 3.1 for Gleason score 3+4), particularly when more aggressive patterns are dominant (OR, 8.7 for Gleason score ≥4+3). Maximum cancer core length (MCCL) increments had an additive effect on the odds of zonal MRI positivity regardless of Gleason grade (OR, 1.24 per millimeter increase), reiterating the need to consider more than grade when addressing MRI-related questions in the prostate. For example, University College London definitions allow for MCCL to be a dominant feature of clinical significance in patients with low Gleason scores and, in our model, a Gleason score of 3+3 could theoretically elicit zonal MRI positivity provided the MCCL is high enough (10). The usefulness of such schemes is corroborated by the finding that University College London definitions were also highly predictive of zonal MRI visibility in mixed models (Fig S2A). When assessing MRI scores of cancerous Barzell zones, we confirmed that high Gleason scores and increased MCCL values are particularly associated with Likert scores of 4 and 5, in contrast to insignificant disease that elicits mainly indeterminate phenotypes (Fig S2B). These conclusions are complementary to previously published work confirming that false negativity is mostly associated with lower grade and small MCCL (11).

The link we found between PIN and false-positive MRI results has been described by others (12). However, PIN is spatially proximal to prostate cancer and, although TPM is the best possible reference standard in a biopsy-naïve population, there is an unavoidable 5%–10% chance of misclassification that could positively bias associations between PIN and false-positive MRI results (10,13,14). Interestingly, our findings on inflammation do not fit the dominant narrative regarding cancer-negative lesions, almost half of which reportedly contain inflammatory foci (15–17). However, we would not immediately interpret our results as evidence against inflammation driving false-positive MRI results because PROMIS did not have MRI-directed sampling, which appears to capture microenvironmental perturbations better than nontargeted needle deployments (18). We also suspect that, although the spatial conformation of immune cells is as important as their count in terms of tissue microstructure, pathologists report inflammation based mostly on the latter.

Finally, we found two “extreme” prostate states captured at TPM: one involving small organs with high csCa burden and the other involving prostates without csCa, where PSA is mainly driven by high organ volume. These extremes and conditions in-between were not age-related. We previously calculated that the MRI volume of prostates with actively surveyed insignificant disease increases by approximately 3.3 mL per year, starting from approximately 50 mL on average at MRI diagnosis (at approximately 63 years of age) (19,20). This starting point is close to the median age and prostate volume of the men without csCa in the current study, but is not compatible with the median prostate volume of men with an overall Gleason score greater than or equal to 4+3 at TPM (which remained at 39 mL despite a slightly greater median age of 64 years). Altogether, these observations raise the question of whether there are two distinct pathologic conditions intercepted by the first PSA-triggered MRI-informed biopsy, which would lead to two clinical scenarios; one is associated with the early detection of csCa in small prostates not undergoing significant age-related growth, and the other is where sampling of already enlarged prostates identifies, on occasion, insignificant disease that either remains stable or progresses over several years while prostatic enlargement continues.

Our study had limitations. First, the inherent coarseness of consensus-based TPM-MRI alignment would explain the unexplored but inevitable discrepancies herein between the prostate-level analyses of PROMIS and our more involved, zone-level examination. This is a direct consequence of PROMIS lacking MRI-directed sampling, which was a necessary compromise to ensure the study investigators were blinded to imaging and pathologic findings. Second, TPM without targeting almost certainly underestimates Gleason pattern 4 and MCCL; deploying a needle toward the lesion center leads to correct grade attribution in 80% of heterogeneous tumors, but deployment in the orientation with the greatest yield is less likely with 5-mm TPM sampling (21). Head-to-head comparisons of MRI targeting and TPM in treatment-naïve men and those with radio-recurrence confirm that, although both biopsy approaches have good detection rates for csCa, MRI targeting captures slightly more high-grade cancers with less needle deployments, whereas TPM depicts more small low-grade lesions (22,23). Third, one could rightly argue that MRI acquisition and interpretation has changed since PROMIS, which is one of the reasons we do not claim our findings are immediately generalizable. However, we suspect our consensus alignment approach could be useful and applicable in other MRI-informed biopsy settings.

In conclusion, the results of this study provide a basis for the MRI signals observed in the prostate. An incremental relationship between cancer burden and prostate MRI visibility was found. Prostatic intraepithelial neoplasia contributed to false-positive MRI findings. Future work will involve a systematic digital histopathologic evaluation of specific microstructural features associated with different MRI endotypes, and how the interaction between different pathologic entities (eg, cancer and inflammation) affects MRI characteristics.

## Supplementary Material

Supplemental Table

## Figures and Tables

**Table 1:** Baseline Characteristics of Included UCLH Nonpilot Participants

Characteristic	Benign (*n* = 52)	Gleason Score 3+3 (*n* = 21)	Gleason Score 3+4 (*n* = 60)	Gleason Score ≥4+3 (*n* = 28)	All Participants (*n* = 161)
Percentage of all participants	33	13	37	17	100
Age (y)	62 (9)	59 (12)	63.5 (10.2)	64 (14.2)	62 (11)
Prostate volume (mL)	55.5 (18.8)	45 (23)	40 (24)	39 (16.2)	44 (25)
PSA level (ng/mL)	4.85 (2.25)	4.8 (2.4)	6.15 (3)	9.35 (2.5)	6 (3.6)
PSA density (ng/mL^2^)	0.09 (0.05)	0.10 (0.06)	0.16 (0.12)	0.25 (0.14)	0.13 (0.12)

Note.–Except where indicated, data are medians, with IQRs in parentheses. Age, prostate volume, prostate-specific antigen (PSA) level, and PSA density are presented for the entire cohort and for each group according to overall Gleason score at transperineal mapping as assigned by the study uropathologists. UCLH = University College London Hospitals.

**Table 2:** Mixed Model of Cancer Burden and Prostate Volume as Predictors of Zonal MRI Visibility in All Nonpilot Participants

Final Model Predictor	Odds Ratio 95% CI	P Value
Gleason score 3+3 (compared with benign)	1.26	0.9, 1.9	.24
Gleason score 3+4 (compared with benign)	3.1	1.9, 4.9	<.001
Gleason score ≥4+3 (compared with benign)	8.7	4.5, 17.0	<.001
ISUP MCCL (per millimeter increase)	1.24	1.15, 1.33	<.001
Binary logarithm of prostate volume (mL)	0.7	0.5, 0.9	.02

Note.–A mixed model with random intercepts for individual participants (*n* = 161) with Gleason grade, MCCL per the ISUP definition, and the binary logarithm of prostate volume (in milliliters) as fixed predictors had the lowest Akaike information criterion and was selected as the final model. The intraclass correlation coefficient was 0.22. ISUP = International Society of Urological Pathology, MCCL = maximum cancer core length.

**Table 3:** Mixed Models of Noncancerous Pathologic Findings as a Determinant of Zonal MRI Positivity in Men who were TPM-Negative

Model and Predictor	Odds Ratio	95% CI	P Value
Full mixed model			
Inflammation	0.8	0.5, 1.5	.49
Prostatic intraepithelial neoplasia	3.2	1.3, 8.1	.01
Atypical small acinar proliferation	1.9	0.7, 5.3	.21
Final mixed model			
Prostatic intraepithelial neoplasia	3.7	1.5, 9.1	.004

Note.–Various combinations of predictors were used in this analysis. In the full mixed model, the presence of chronic inflammation or atypical small acinar proliferation did not increase the odds of declaring a zone MRI-visible (see also Table S1). A reduced mixed model with random intercepts for individual participants and only prostatic intraepithelial neoplasia as a fixed predictor had the lowest Akaike information criterion and, thus, was selected. The intraclass correlation coefficient was 0.49. There were 52 men (1031 Barzell zones) with negative TPM results. TPM = transperineal mapping.

## Data Availability

Data analyzed during the study were provided by a third party. Requests for data should be directed to the provider indicated in the Acknowledgments.

## Summary

In this analysis, there was an incremental relationship between prostate cancer burden and the likelihood of a positive MRI signal.

### Key Results

■ In 161 men from the Prostate MRI Imaging Study, prostate zones that contained Gleason score 3+4 cancer were three times more likely to be MRI-positive than benign zones (odds ratio [OR], 3.1; *P* < .001), and almost nine times if they had a Gleason score greater than or equal to 4+3 (OR, 8.7; *P* < .001).
■ Increasing maximum cancer core length raised the odds of a cancerous zone being MRI-positive (OR, 1.24 per millimeter increase; *P* < .001).
■ In cancer-free prostates, zones containing prostatic intraepithelial neoplasia were more likely to be MRI-positive (OR, 3.7; *P* = .004).

## Abbreviations

csCa: clinically significant cancer
MCCL: maxim um cancer core length
OR: odds ratio
PIn: prostatic intraepithelial neoplasia
PROMIS: Prostate MRI Imaging Study
PSA: prostate-specific antigen
TPM: transperineal mapping
