# Supplementary material for: Regional Histopathology and Prostate MRI Positivity: A Secondary Analysis of the PROMIS Trial
Source: Radiology. Author manuscript; Available in PMC 2023 Jun 20. (PMC7614676; doi:10.1148/radiol.220762)
Supplement: Supplemental Table [file EMS174630-supplement-Supplemental_Table.pdf]

©RSNA, 2022
10.1148/radiol.220762

Table S1

AIC-based Mixed Model Selection for True (Top Table) and False (Bottom Table) MRI Positivity

Model	Predictors	AIC
Null GLM	Intercept only	3086.948
Null Mixed	Random intercept only	2924.294
Mixed 1	Gleason	2604.279
Mixed 2	Gleason + MCCL	2567.339
Mixed 3 (final)	Gleason + MCCL + log_2vol	2563.581

Model	Predictors	AIC
Null GLM	Intercept only	753.9745
Null Mixed	Random intercept only	650.1202
Mixed 1	Inflammation + PIN + ASAP	645.1227
Mixed 2	Inflammation + PIN	644.5964
Mixed 3 (final)	PIN	643.1035

Note.—Akaike information criterion (AIC) values for all fitted models are presented. Mixed models performed better than generalized linear models (GLM) with fixed effects only, while the AIC of the two selected mixed models were the lowest. The addition of interactions led to model nonconvergence, so no interaction terms were included. MCCL = maximum cancer core length, PIN = prostatic intraepithelial neoplasia, ASAP = atypical small acinar proliferation, log_2vol = binary logarithm of prostate volume in mL.
